# Results of a Prospective Trial to Compare ^68^Ga-DOTA-TATE with SiPM-Based PET/CT vs. Conventional PET/CT in Patients with Neuroendocrine Tumors

**DOI:** 10.3390/diagnostics11060992

**Published:** 2021-05-30

**Authors:** Lucia Baratto, Akira Toriihara, Negin Hatami, Carina M. Aparici, Guido Davidzon, Craig S. Levin, Andrei Iagaru

**Affiliations:** 1 Division of Nuclear Medicine and Molecular Imaging, Department of Radiology, Stanford University, Stanford, CA 94035, USA; neginh@stanford.edu (N.H.); drmari@stanford.edu (C.M.A.); gdavidzon@stanford.edu (G.D.); aiagaru@stanford.edu (A.I.); 2 PET Imaging Center, Asahi General Hospital, Asahi 289-1101, Japan; 3 Molecular Imaging Program, Department of Radiology, Stanford University, Stanford, CA 94305, USA; cslevin@stanford.edu

**Keywords:** ^68^Ga-DOTA-TATE PET, silicon photomultiplier, PET/CT, neuroendocrine tumor

## Abstract

We prospectively enrolled patients with neuroendocrine tumors (NETs). They underwent a single ^68^Ga-DOTA-TATE injection followed by dual imaging and were randomly scanned using first either the conventional or the silicon photomultiplier (SiPM) positron emission tomography/computed tomography (PET/CT), followed by imaging using the other system. A total of 94 patients, 44 men and 50 women, between 35 and 91 years old (mean ± SD: 63 ± 11.2), were enrolled. Fifty-two out of ninety-four participants underwent SiPM PET/CT first and a total of 162 lesions were detected using both scanners. Forty-two out of ninety-four participants underwent conventional PET/CT first and a total of 108 lesions were detected using both scanners. Regardless of whether SiPM-based PET/CT was used first or second, maximum standardized uptake value (SUV_max_) of lesions measured on SiPM was on average 20% higher when comparing two scanners with all enrolled patients, and the difference was statistically significant. SiPM-based PET/CT detected 19 more lesions in 13 patients compared with conventional PET/CT. No lesions were only identified by conventional PET/CT. In conclusion, we observed higher SUV_max_ for lesions measured from SiPM PET/CT compared with conventional PET/CT regardless of the order of the scans. SiPM PET/CT allowed for identification of more lesions than conventional PET/CT. While delayed imaging can lead to higher SUV_max_ in cancer lesions, in the series of lesions identified when SiPM PET/CT was used first, this was not the case; therefore, the data suggest superior performance of the SiPM PET/CT scanner in visualizing and quantifying lesions.

## 1. Introduction

Positron emission tomography/computed tomography (PET/CT) plays a critical role in the management of oncologic patients. International guidelines recommend the use of PET/CT for the detection of primary as well as metastatic disease for many different tumors, depending on the tumor itself and on the specific radiotracer [1,2,3,4]. The use of PET/CT using ^68^Ga-DOTA-conjugated peptides is recommended to determine the status of somatostatin (SST) receptors in patients with neuroendocrine tumors (NETs) for staging and restaging purposes, as well as to select patients eligible for SST receptor radionuclide therapy [3,4].

To improve patient care, we need to develop PET/CT technology with better spatial resolution, reduction of noise, and higher accuracy of the quantitative data.

Photosensors are key elements of PET scanners, they adjust the light output of the system and convert it into a proportional electrical signal based on the amount of light the system senses at a particular location. The most used photosensor in gamma ray detectors is the photomultiplier tube (PMT) since it has advantageous characteristics: fast rise time, high quantum efficiency (QE), and relatively high gain [5]. PMTs have been used in previous generation PET scanners, and they are essential components in time of flight (ToF) PET detectors. However, they also have some limitations that affect, in particular, reconstructed image signal-to-noise ratio (SNR) [6,7,8].

Over the last decade, new photosensors, the silicon photomultipliers (SiPMs), were introduced into commercial PET systems [7]. We previously reported the performance of a SiPM PET/CT scanner (GE Discovery Molecular Insights—DMI PET/CT, GE Healthcare, Waukesha, WI, USA) [8] and compared it to conventional PET/CT scanners (GE Discovery 600 and GE Discovery 690) in cancer patients undergoing 2-Deoxy-2-[^18^F]fluoroglucose (FDG) imaging (9). At the time of that study, the DMI PET/CT had not been approved by the Food and Drug Administration (FDA); therefore, the standard scanners were always used first, followed by SiPM-based PET/CT [9].

Here we prospectively compared the DMI and D690 scanners in patients with NETs following a single injection of ^68^Ga-DOTATATE to evaluate differences in quantitative and semi-quantitative measurements.

## 2. Materials and Methods

### 2.1. Participants

Patients with NETs were prospectively enrolled in this protocol between April 2017 and April 2019 and randomized to be scanned first using DMI followed by D690 or to be scanned first using D690 followed by DMI. The study was approved by the Stanford University institutional review board, and all participants signed an informed consent.

### 2.2. D690 PET/CT Protocol

The CT scan was obtained for attenuation correction and anatomical localization using 120 kV, “smart” modulating mA and a 512 × 512 matrix size. Thereafter, a whole-body (vertex to mid-thighs) PET scan was acquired in 3D mode with ToF enabled. D690 (GE Discovery 690, GE Healthcare, Waukesha, WI, USA) has an axial field of view of 15.7 cm. Each field of view contains 47 slices (3.27 mm), and the overlap between bed positions was set to 11 slices (23%). The acquisition time was 3 min per bed position. PET data were corrected using the segmented attenuation data of the CT scan and reconstructed using the vendor-recommended reconstruction protocol with an ordered subset expectation maximization (OSEM) with 2 iterations and 24 subsets. When performed as second scan, a low-dose CT (10 mA) was obtained for attenuation correction and anatomic localization to minimize radiation exposure to participants.

### 2.3. DMI PET/CT Protocol

The CT scan was obtained for attenuation correction and anatomical localization using 120 kV, “smart” modulating mA and a 512 × 512 matrix size. Thereafter, a whole-body (vertex to mid-thighs) PET scan was acquired in 3D mode with time-of-flight (ToF) enabled. DMI (GE Discovery Molecular Insights—DMI PET/CT, GE Healthcare, Waukesha, WI, USA) has an axial field of view of 20 cm. Each field of view contains 71 slices (2.79 mm), and the overlap between bed positions was set to 17 slices (24%). The acquisition time was 3 min per bed position. PET data were corrected using the segmented attenuation data of the CT scan. Although reconstruction using the block sequential regularized expectation maximization (BSREM) protocol [10] was available, we reconstructed data using OSEM to allow for direct comparison with the D690 data. When performed as second scan, a low-dose CT (10 mA) was obtained for attenuation correction and anatomic localization to minimize radiation exposure to participants.

### 2.4. Image Analysis

Scans were independently reviewed and analyzed by two nuclear medicine physicians (LB and AT, with 9 and 11 years of experience, respectively) using Advantage Workstation (GE Healthcare, Waukesha, WI, USA). Any disagreements were resolved by consensus. Paired scans (SiPM and conventional PET/CT) were displayed using a fixed SUV scale (threshold 50%) and color table. For the evaluation of semiquantitative metrics (maximum and mean standardized uptake values—SUV_max_ and SUV_mean_) images were displayed in a trans-axial view and a 3D region-of-interest (ROI) was placed in up to six detected lesions with the highest uptake. All lesions recorded on the first scan were matched to lesions on the second scan. We also recorded all the lesions detected only using SiPM scanner. A 2D circular ROI was used to measure uptake in normal organs (pituitary, parotid, aortic arch, lung, liver, spleen, adrenals, gluteal muscle, and gluteal fat). Location of ROIs for single background organs was decided by consensus between the two readers prior to the start of the analysis. Size of ROIs changed depending on the structure of interest. The ROIs for the liver were drawn excluding any lesions. The ROIs for the mediastinal blood pool were placed on the aortic arch. Contrast recovery (CR) was calculated as the ratio between lesional SUV_max_ and blood pool SUV_mean_. Measurements from both the SiPM and the conventional PET/CT were recorded from images reconstructed with OSEM and TOF.

### 2.5. Statistical Analysis

Statistical analysis was performed with SPSS v26 (SPSS Inc., Chicago, IL, USA) and STATA RELASE 14.2 (Stata Corp LP, College Station, TX, USA). Continuous data are presented as mean ± standard deviation (SD), minimum-maximum values, and frequencies (%). Since the data did not follow a normal distribution, we performed the Wilcoxon signed ranked test to compare SUV_max_ differences between the two scanners. A *p*-value < 0.05 was considered significant. To evaluate the agreement between scanners on background organs, the intraclass correlation was calculated through a mixed effects model with clustering within patients, while taking time into account. The linear regression analysis was applied for the evaluation of SUV_max_ differences, while controlling for the time delay between the two scanners.

## 3. Results

### 3.1. Patients’ Characteristics and PET Findings for the Entire Cohort

**Figure 1:**
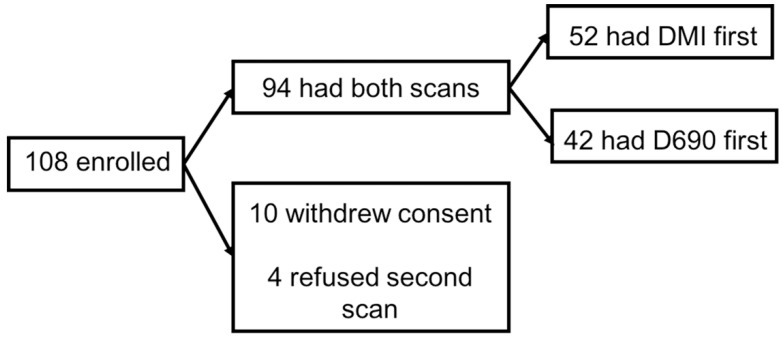
Flowchart of the study population.

One hundred and eight patients with NETs were prospectively enrolled in this protocol between April 2017 and April 2019. However, 14 of the 108 patients were excluded after enrollment: 10 withdrew their consent and 4 decided to not undergo the second scan. Figure 1 shows a flowchart of the enrolled participants. Therefore, data from 94 participants, 44 men and 50 women, between 35 and 91 years old (mean ± SD: 63 ± 11.2), were analyzed. The body mass index (BMI) ranged from 17.6 to 44.8 (mean ± SD: 27.9 ± 5.7). Among the participants, 29 of 94 (31%) were referred for initial treatment strategy (formerly diagnosis and initial staging), while 65 of the 94 participants (69%) were referred for subsequent treatment strategy (including treatment monitoring, restaging, and detection of suspected recurrence). This classification is based on the National Coverage Determination for PET for Oncologic Conditions from the Centers for Medicare & Medicaid Services [11] Patients’ characteristics are described in Supplementary Material Table S1). ^68^Ga-DOTA-TATE dosage ranged from 3.5 to 7.3 mCi (mean ± SD: 5.3 ± 0.8 mCi). The time from injection to the scan ranged from 42.9 to 93.5 min (mean ± SD: 61.3 ± 11) and from 62.5 to 143.6 min (mean ± SD: 88.1 ± 16.0) for the first and the second scan, respectively. The delay time between the two scans ranged from 17.6 to 61.8 min (mean ± SD: 26.8 ± 9.5). 

Agreement analysis showed that the two scanners were comparable in terms of normal background organs uptake, independent from the time delay (*p* > 0.05). These results are shown in Table 1. 

For semi-quantitative analysis, we measured up to 6 lesions with the highest uptake and identified by both scanners: a total of 270 NET lesions in 70 out of 94 (74%) patients were detected by both scanners. SUV_max_ ranged from 1.2 to 225 (mean ± SD: 31.4 ± 31.6) and from 0.9 to 234.3 (mean ± SD: 29.4 ± 29.5) for SiPM and conventional PET/CT, respectively. The difference in SUV_max_ measurements between the two scanners was statistically significant (*p* < 0.001). 

### 3.2. Results When SiPM PET/CT Was Used First Followed by Conventional PET/CT

Fifty-two patients, twenty-three men and twenty-nine women, 43 to 85 years old (mean ± SD: 63.3 ± 10.4) underwent SiPM PET/CT first, followed by conventional PET/CT. BMI ranged from 18.2 to 44.8 (mean ± SD: 28.4 ± 5.8). ^68^Ga-DOTA-TATE dosage ranged from 3.5 to 6.7 mCi (mean ± SD: 5.1 ± 0.8). Imaging started between 46.2 and 93.5 min (mean ± SD: 63.3 ± 10.7) and between 66.6 and 143.6 min (mean ± SD: 88.7 ± 16.7) after injection for SiPM and conventional PET/CT, respectively. The delay time between the two scans ranged between 17.6 and 61.8 min (mean ± SD: 25.4 ± 9.7). 

A total of 162 lesions were detected in 38 patients (40 lymph nodes, 67 hepatic nodules, 16 bone lesions, 11 pancreatic nodules, and 28 other sites of disease) using both scanners. SUV_max_ for these 162 lesions ranged from 3.2 to 118.8 (mean ± SD: 28.7 ± 19.6) and from 2.5 to 109.0 (mean ± SD: 27.6 ± 19) for SiPM and conventional PET/CT, respectively; the difference in SUV_max_ trended towards statistical significance (*p* = 0.049).

When SUV_max_ was analyzed per single organ, the average measurements between the two scanners were significantly different for bone lesions (*p* = 0.03), trending towards significance for lymph nodes (*p =* 0.052) and not significant for liver, pancreas, and other sites of disease. Results are shown in Table 2. 

CR was on average higher for SiPM-based compared with conventional PET/CT and ranged from 3.5 to 127.1 (mean ± SD: 38.9 ± 32.1) and from 4.1 to 137.4 (mean ± SD: 35.4 ± 30), respectively; this difference was not statistically significant (*p =* 0.055). 

Compared with the conventional PET/CT, SiPM PET/CT identified 12 more ^68^Ga-DOTA-TATE avid lesions in 8 patients (5 lymph nodes, 5 hepatic nodules, 2 bone lesions). These lesions had SUV_max_ measurements ranging from 1.7 to 13.3. (mean ± SD: 7.1 ± 4). There were no lesions seen only on conventional PET/CT.

**Figure 2:**
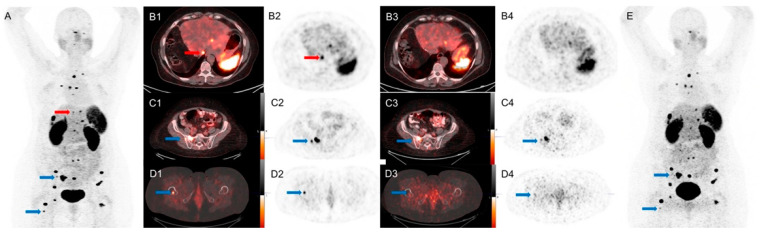
A 65-year-old woman with metastatic NET of unknown primary, referred for subsequent treatment strategy after chemotherapy. SiPM-based PET/CT was performed first at 72 min after injection of 4.2 mCi of ^68^Ga-DOTA-TATE. Conventional PET/CT was performed second at 96 min after injection. Red arrows mark an additional lesion seen in the dome of the liver only on SiPM-based PET/CT (**A**,**B1**), not on conventional PET/CT (**B3**,**B4**,**E**). Blue arrows mark bone lesions seen on conventional PET/CT (**C3**,**C4**,**D3**,**D4**,**E**) but more conspicuous on SiPM-based PET/CT (**A**,**C1**,**C2**,**D1**,**D2**).

An example is shown in Figure 2.

### 3.3. Results When Conventional PET/CT Was Used First Followed by SiPM

Forty-two patients, twenty-one men and twenty-one women, between 35 and 91 years old (mean ± SD: 62.8 ± 12.2) underwent conventional PET/CT first, followed by SiPM PET/CT. BMI ranged from 17.6 to 39.8 (mean ± SD: 27.2 ± 5.5). ^68^Ga-DOTA-TATE dosage ranged from 3.6 to 7.3 mCi (mean ± SD: 5.5 ± 0.9 mCi). Imaging started between 42.9 and 90.7 min (mean ± SD: 58.8 ± 10.9) and between 62.5 and 138.4 min (mean ± SD: 87.4 ± 15.1) after injection for conventional and SiPM PET/CT, respectively. The delay time between the two scans ranged between 17.0 and 50.2 min (mean ± SD: 28.5 ± 9).

A total of 108 lesions were detected in 32 patients (17 lymph nodes, 55 hepatic nodules, 8 bone lesions, 13 pancreatic nodules, and 15 other site of disease) using both scanners. SUV_max_ for these 108 lesions ranged from 0.9 to 234.3 (mean ± SD: 32 ± 40.4) and from 1.2 to 225 (mean ± SD: 35.4 ± 43.7) for conventional and SiPM PET/CT, respectively. This difference in SUV_max_ measurements was statistically significant (*p* < 0.001).

Since in this sub-cohort of patients, the SiPM-based system was used for the second scan, we attempted to compare the two scanners after controlling for the time difference. A linear regression analysis was performed, and a weak association was found between acquisition time delay and the SUV_max_ difference (*R*^2^ = 0.18).

When SUV_max_ measurements were analyzed per single organ lesions, the average values between the two scanners were significantly different for hepatic and pancreatic lesions (*p* < 0.001 and *p* < 0.003, respectively) but not for lymph nodes, bone lesions, and other site of disease. Results are shown in Table 3.

CR ranged from 1.3 to 262 (mean ± SD: 41.7 ± 59.2) and from 1 to 227.6 (mean ± SD: 44.8 ± 56.6) for conventional and SiPM-based PET/CT data, respectively. This difference was statistically significant (*p* < 0.01).

Compared with the conventional PET/CT, SiPM PET/CT identified 7 more ^68^Ga-DOTA-TATE avid lesions in 5 patients (4 lymph nodes, 2 hepatic nodules, and 1 bone lesion). These lesions had SUV_max_ measurements ranging from 3.7 to 8.3 (mean ± SD: 5.6 ± 1.6). There were no lesions seen only on conventional PET/CT.

**Figure 3:**
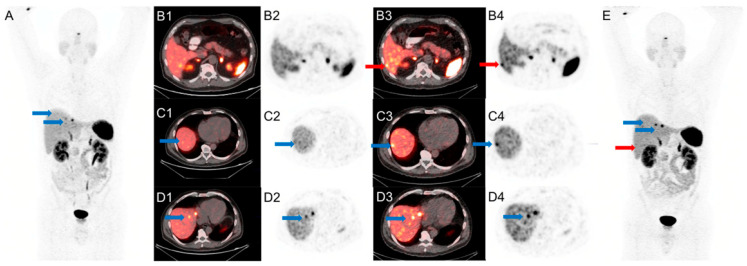
A 59-year-old man with metastatic small bowel NET, referred for subsequent treatment strategy after small bowel resection. Conventional PET/CT was performed first at 85 min after injection of 4.5 mCi of ^68^Ga-DOTA-TATE. SiPM-based PET/CT was performed second at 110 min after injection. Red arrows mark an additional lesion seen in the right lobe of the liver only on SiPM-based PET/CT (**B3**,**B4**,**E**) not on conventional PET/CT (**A**,**B1**,**B2**). Blue arrows mark liver lesions seen on conventional PET/CT (**A**,**C1**,**C2**,**D1**,**D2**) but more conspicuous on SiPM-based PET/CT (**C3**,**C4**,**D3**,**D4**,**E**).

An example is shown in Figure 3.

Characteristics of the patients who had lesions identified only using SiPM PET/CT are shown in Table 4.

## 4. Discussion

Our results suggest better performance of SiPM PET/CT compared with conventional PET/CT regardless of the order of the scans. This was noted both in terms of overall detection and semi-quantitative measurements. Average SUV_max_ values were consistently higher for the SiPM-based system images than for conventional PET/CT. SiPM PET/CT detected 19 more lesions.

Other studies evaluated the difference between SiPM-based vs. conventional PET/CT scanners, reporting better performance of the SiPM-based systems [9,12,13]. Fuentes-Ocampo et al. [12] evaluated 100 patients with either ^18^F-FDG or ^18^F-Choline PET/CT and compared SiPM-based vs. conventional PET/CT scanners in terms of SUV_max_ of the target lesions and of mediastinal blood pool and liver as background organs. They reported that semi-quantitative values from the SiPM-based PET/CT were always higher than from the conventional ones, regardless the order of the scan; the difference was significant for detected lesions and blood pool but not for liver. In our study, we recorded higher SUV_max_ values when using SiPM-based PET/CT, both for the lesions analyzed and for single organs analysis. SUV_max_ was higher when SiPM PET/CT was performed as second scan independent from the time delay (*R*^2^ = 0.18), similar to what Ocampo et al. reported in their study (*R*^2^ = 0.29). We calculated CR, and the average values from SiPM-based PET/CT were higher. These findings may be explained by the better spatial resolution, photon sensitivity, and time-of-flight SNR gain for this SiPM-based PET system compared with the conventional PET/CT system used (average NEMA sensitivity of 13.9 cps/kBq vs. 7.5 cps/kBq for DMI and D690, respectively) [8].

López-Mora et al. [13] compared image quality and overall detection rate in 100 oncological patients imaged with ^18^F-FDG or ^18^F-Choline PET/CT between SiPM-based and conventional PET/CT. Their results indicate improved image quality when using SiPM-based PET/CT and more lesions detected by the SiPM-based PET/CT in 22 patients. Additionally, disease stage was modified in 7 out of those 22 patients based on findings from SiPM-based PET/CT. In our study, SiPM-based PET/CT identified 19 more lesions in 13 patients compared with conventional PET/CT, and most of them were lymph nodes metastases, which have important implications for treatment decisions and can be difficult to identify by conventional structural imaging [14]. However, disease stage did not change in our cohort of patients, most likely due to the advanced disease stage of most NET patients in our cohort. This is not unexpected in a tertiary medical center.

We previously reported the higher performance of SiPM-based PET/CT in a cohort of participants who underwent FDG PET/CT for oncological indications [9]. They were scanned with either D600 or D690 PET/CT as a routine clinical examination and underwent the DMI scan immediately after for research purpose. At that time, the order of the scanners could not be randomized since DMI had not yet been approved by the Food and Drug Administration. Nevertheless, an increase in SUV_max_ was reported. Comparison between background organs showed that no significant differences were registered between the two scanners, confirming the results in the previous study [9].

This study has limitations: lesions detected only by the SiPM PET/CT scanner were not confirmed with biopsy, so some may have been false positive findings. Moreover, the block sequential regularized expectation maximization (BSREM) algorithm (Q.Clear^®^, GE Healthcare) was not used to reconstruct SiPM PET/CT images. BSREM controls noise at higher iterations, and we have shown that it improves image quality compared with the ordered subsets expectation maximization algorithm (OSEM) [15]. Nevertheless, we decided not to use it here since we could not apply it to reconstruct data from the conventional PET/CT (system incompatibility), and the main goal of the study was the comparison between the two scanners, without choice of reconstructions algorithm introducing bias in that comparison.

## 5. Conclusions

The SUV_max_ measurements were higher in lesions detected by SiPM than by conventional PET/CT, regardless of the order of the scan. There was a better CR in images acquired using SiPM PET/CT. Nineteen lesions in thirteen patients were only identified using the SiPM PET/CT. The results indicate that SiPM PET/CT has superior performance compared with a conventional PET/CT scanner.

## Figures and Tables

**Table 1:** SUV_mean_ measurements in background organs.

Organs	SiPM PET/CT SUV_mean_ (Mean ± SD)	Conventional PET/CT SUV_mean_ (Mean ± SD)	Difference of SUV_mean_ (mean ± SD)	95% CI	ICC
Pituitary	7.87 ± 3.91	5.80 ± 3.13	2.07 ± 2.03	0.075–0.823	0.379
Parotid	2.22 ± 1.30	2.18 ± 1.32	0.04 ± 0.38	0.000–0.990	0.010
Aortic arch	1.05 ± 0.37	1.07 ± 0.45	−0.02 ± 0.40	0.548–0.977	0.878
Lung	0.46 ± 0.41	0.47 ± 0.42	−0.012 ± 0.15	0.899–0.997	0.982
Liver	4.70 ± 2.26	5.17 ± 2.64	−0.47 ± 1.17	0.753–0.956	0.891
Spleen	17.14 ± 6.63	17.64 ± 7.54	−0.50 ± 2.85	0.851–0.941	0.905
Adrenals	9.52 ± 4.88	9.24 ± 4.90	0.27 ± 2.46	0.828–0.912	0.876
Gluteal muscle	0.58 ± 0.18	0.60 ± 0.27	−0.01 ± 0.21	0.805–0.995	0.966
Gluteal fat	0.35 ± 0.14	0.35 ± 0.19	0.005 ± 1.10	0.948–0.999	0.992

CI: confidence interval. ICC: intraclass correlation coefficient. The agreement analysis investigated the consistency between SiPM and conventional PET/CT scanners in different background locations. ICC was calculated through mixed effects model with clustering within patients, while considering time. The correlation results are shown in the table. The agreement was solid between scanners (all correlations are around 0.9) except for parotid and pituitary. Time of study relative to injection time did not matter for all organs (all *p* values > 0.05).

**Table 2:** Single organ analysis for patients who underwent SiPM PET/CT as first scan (*n* = 52).

Organs	SiPM (Mean ± SD)	Conventional PET (Mean ± SD)	*p* Value	*n*
All	28.7 ± 19.6 (range: 3.2–118.8)	27.6 ± 19.1 (range: 2.5–109.0)	0.049	162
Lymph nodes	28.6 ± 14.9 (range: 3.2–77.5)	27.6 ± 16.5 (range: 3.6–68.4)	0.052	40
Liver	26.9 ± 14.2 (range: 4.8–59.6)	26.2 ± 14.5 (range: 5–71)	0.619	67
Bone	34.2 ± 29.4 (range: 3.8–88.3)	27.1 ± 23.1 (range: 2.5–69.6)	0.034	16
Pancreas	34.7 ± 18.9 (range: 8.1–67)	33.9 ± 22.8 (range: 3.6–83.4)	0.286	11
Other	27.6 ± 28.6 (range: 3.6–118.8)	28.8 ± 27.7 (range: 34.1–109)	0.419	28

*n* = total number of lesions. Other: 6 peritoneum, 1 duodenum, 4 colon, 1 appendix, 4 small bowel, 3 lung, 3 muscles, 1 pericardium, 1 thoracic wall, 1 renal pelvis, 1 stomach wall, 1 parapharyngeal mass, 1 optic canal. Average SiPM PET/CT SUV_max_ was higher for all organs analyzed (except for other sites of lesions), but the difference was significant only for bone lesions.

**Table 3:** Single organ analysis for patients who underwent conventional PET/CT as first scan (*n* = 42).

Organs	Conventional PET (Mean ± SD)	SiPM (Mean ± SD)	*p* Value	*n*
All	32 ± 40.4 (range: 0.9–234.3)	35.4 ± 43.7 (range: 1.2–225)	<0.001	108
Lymph nodes	46.6 ± 62.1 (range: 2.3–234.3)	49 ± 62.4 (range: 2.4–215.7)	0.163	17
Liver	30.6 ± 23.8 (range: 7.19–45.6)	34.4 ± 27.8 (range: 6.81–42.8)	<0.001	55
Bone	64.1 ± 82.2 (range: 2.1–209.1)	72.2 ± 92.6 (range: 2.2–225)	0.123	8
Pancreas	26.2 ± 33.3 (range: 5–124.9)	29 ± 34.2 (range: 6.5–126.5)	0.003	13
Other	8.5 ± 7.3 (range: 0.9–21.7)	9.3 ± 8.2 (range: 1.2–22.4)	0.280	15

*n* = total number of lesions. Other: 1 peritoneum, 2 breast, 1 spleen, 1 atrium, 7 lung, 2 pleural, 1 muscle. Average SiPM PET/CT SUV_max_ was higher for all organs analyzed, but the difference was significant only for liver and pancreatic lesions.

**Table 4:** Characteristics of the 13 participants who had lesions detected only with SiPM PET/CT.

Age	Sex	Referral Category	First Scan	Delay Time (min)	Lesions Detected by SiPM PET/CT	Lesions Detected by Conventional PET/CT	Lesion Location
50	F	Restaging	SiPM	19.1	6	4	Lymph nodes
76	F	Restaging	SiPM	19.2	7	6	Liver
64	M	Staging	SiPM	20.3	8	6	Liver
58	M	Surveillance	SiPM	22.9	7	6	Liver
47	F	Restaging	SiPM	18.9	9	6	Liver
82	M	Staging	SiPM	22.1	2	1	Liver, bone
67	M	Staging	SiPM	23.4	3	2	Lymph nodes
74	F	Staging	SiPM	21.3	4	3	Lymph nodes
49	F	Staging	Conventional	43.8	8	6	Bone, Lymph nodes
52	M	Surveillance	Conventional	35.7	2	1	Lymph nodes
54	F	Staging	Conventional	19.6	7	6	Liver
41	M	Restaging	Conventional	46.8	7	6	Liver
72	M	Restaging	Conventional	25.5	8	6	Lymph nodes

## Data Availability

Not applicable.

## Supplementary Materials

The following are available online at https://www.mdpi.com/article/10.3390/diagnostics11060992/s1, Table S1: Patients’ Characteristics.
