# Supplementary material for: Results of a Prospective Trial to Compare 68Ga-DOTA-TATE with SiPM-Based PET/CT vs. Conventional PET/CT in Patients with Neuroendocrine Tumors
Source: Diagnostics (Basel). 2021 May 30;11(6):992. doi: 10.3390/diagnostics11060992 (PMC8228776; doi:10.3390/diagnostics11060992)
Supplement: Supplementary file 1 [file diagnostics-11-00992-s001.zip › diagnostics-1239138-supplementary.pdf]

Supplementary Table S1: Patients' characteristics.

#	Age	Sex	Primary location	Tumor characteristics	Previous Treatments	Purpose of PET/CT
1	68	M	Adrenal	METs	Surgery	Subsequent treatment strategy
2	52	F	Unknown Primary	NA	None	Subsequent treatment strategy
3	71	F	Unknown Primary	NA	None	Subsequent treatment strategy
4	70	M	Small Bowel	WD, grade 2, Ki-67<2%, NF, stage IV	Surgery	Subsequent treatment strategy
5	64	M	Pancreas	WD, grade 1, stage IIB	None	Initial treatment strategy
6	66	F	Duodenum	stage IV	Surgery	Subsequent treatment strategy
7	62	F	Unknown Primary	NA	None	Subsequent treatment strategy
8	49	F	Small Bowel	WD, grade 2, Ki-67 3.8%, stage IV, NF	Surgery	Subsequent treatment strategy
9	65	M	Rectum	WD, grade 1, Ki-67 2 %, pT1bNx, NF	Surgery	Initial treatment strategy
10	50	F	Lung	grade 2, Ki-67 8%, T1a N0 MX, NF	None	Subsequent treatment strategy
11	71	F	Small Bowel	WD, stage IV, F	Surgery + Octreotide	Subsequent treatment strategy
12	72	F	Cecum/ileum	stage IV, F	Surgery + Octreotide	Subsequent treatment strategy
13	60	F	Adrenal	WD, grade 1, Ki-67 <1%, pT1 pN0, MEN-1, F	None	Subsequent treatment strategy
14	49	M	Pancreas	WD, grade 2, Ki-67 3%, pT2N1, NF	Surgery	Subsequent treatment strategy
15	68	M	Small Bowel	WD, grade 1, Ki-67 <1%, pT3N0, F	Surgery	Subsequent treatment strategy
16	76	F	Ileo	stage IV, F	Octreotide	Subsequent treatment strategy
17	49	F	Pancreas	WD, grade 2, Ki-67 5%, stage IV, NF	Surgery	Initial treatment strategy
18	79	F	Unknown Primary	NA	None	Initial treatment strategy
19	79	M	Gallbladder	PD, grade 2, Ki-67 8-9%, stage IV, F	Chemotherapy	Initial treatment strategy
20	35	F	Unknown Primary	NA	None	Subsequent treatment strategy
21	78	M	Unknown Primary	NA	None	Initial treatment strategy
22	66	F	Unknown Primary	NA	Surgery + Chemotherapy	Subsequent treatment strategy

23	75	F	Small bowel	WD, grade 2, Ki-67 6.4%, stage IV, F	Octreotide	Initial treatment strategy
24	60	M	Colon	WD, grade 1, Ki-67 1.4%, stage IV, NF	None	Subsequent treatment strategy
25	80	F	Lung	WD, grade 1, Ki-67<5%, T1, NF	None	Subsequent treatment strategy
26	51	F	Small bowel	NA	Surgery	Subsequent treatment strategy
27	58	M	Unknown Primary	NA	None	Initial treatment strategy
28	72	M	Lung	NA	None	Subsequent treatment strategy
29	68	F	Lung	WD, grade 1, pT1 pNX, NF	Surgery + Radiotherapy	Subsequent treatment strategy
30	43	F	Colon	MD, grade 2, Ki-67 20%, stage IIA, F	None	Subsequent treatment strategy
31	52	M	Pancreas	WD, grade 2, Ki-67 2%, pT1pN0, NF	Surgery	Subsequent treatment strategy
32	53	M	Thyroid	NA	Surgery	Subsequent treatment strategy
33	67	M	Liver	WD, grade 1, Ki-67 2%, stage IV, NF	Surgery	Subsequent treatment strategy
34	54	F	Small Bowel	WD, grade 2, Ki 67 3.4%, stage IV, F	None	Initial treatment strategy
35	64	M	Unknown Primary	WD, grade 1, stage IV, NF	None	Initial treatment strategy
36	74	F	Lung	WD, stage IV, NF	None	Initial treatment strategy
37	55	F	Small Bowel	WD, grade 1, Ki 67 <1%, pT4 pN1, F	Octreotide	Subsequent treatment strategy
38	46	F	Pituitary	WD, grade1, Ki 67 <1%, F	Surgery + Radiotherapy	Subsequent treatment strategy
39	59	M	Unknown Primary	WD, grade 1, Ki 67 0.6%, stage 3, NF	None	Initial treatment strategy
40	58	M	Small Bowel	NA	Surgery	Subsequent treatment strategy
41	72	M	Bronchus	WD, grade 2, Ki 67 3.7%, stage IV, NF	None	Subsequent treatment strategy
42	65	F	Pancreas	NA	Surgery	Subsequent treatment strategy
43	64	F	Adrenal	METs, F	None	Subsequent treatment strategy
44	64	F	Small Bowel	stage IV, F	Surgery	Subsequent treatment strategy
45	52	M	Pancreas	WD, grade 2, Ki 67 10%, stage IV, NF	None	Subsequent treatment strategy
46	75	M	Unknown Primary	NA	Surgery	Subsequent treatment

						strategy
47	66	F	Pancreas	NA	Surgery	Subsequent treatment strategy
48	50	F	Unknown Primary	WD, grade 2, Ki 67 2.8%, pT1N0, F	None	Initial treatment strategy
49	41	M	Small Bowel	WD, grade 2, Ki 67 9%, pT1N0, NF	Surgery	Subsequent treatment strategy
50	75	M	Unknown Primary	WD, grade 2, stage IV, NF	Surgery + Chemotherapy	Subsequent treatment strategy
51	64	M	Unknown Primary	WD, grade 1, stage IV, NF	None	Subsequent treatment strategy
52	56	F	Rectum	WD, grade 1, Ki 67 <1%, stage II, NF	Surgery	Subsequent treatment strategy
53	72	M	Small Bowel	NA	Surgery	Subsequent treatment strategy
54	74	M	Unknown Primary	NA	None	Subsequent treatment strategy
55	50	F	Unknown Primary	WD, NF	None	Subsequent treatment strategy
56	50	M	Lung	grade 2, Ki 67 20%, stage IV, NF	None	Subsequent treatment strategy
57	50	M	Unknown Primary	F	None	Subsequent treatment strategy
58	60	F	Unknown Primary	NA	None	Initial treatment strategy
59	59	M	Small Bowel	WD, grade 1, Ki 67 < 0.1%, stage IV, NF	Surgery	Subsequent treatment strategy
60	50	M	Stomach	WD, grade 2, stage II, NF	None	Initial treatment strategy
61	47	F	Stomach	NA	Surgery	Subsequent treatment strategy
62	47	F	Lung	PR, grade 2, Ki 67 6%, stage IV, NF	Surgery	Subsequent treatment strategy
63	70	F	Unknown Primary	NA	None	Subsequent treatment strategy
64	66	F	Unknown Primary	WD, stage IV, NF	None	Initial treatment strategy
65	63	M	Pancreas	NA	Surgery	Subsequent treatment strategy
66	68	F	Lung	WD, grade 2, Ki 67 15%, stage IV, NF	Surgery	Subsequent treatment strategy
67	73	F	Colon	WD, grade 1, Ki 67 1%, stage II	Surgery	Subsequent treatment strategy
68	82	M	Esophagogastric Junction	NA	None	Initial treatment strategy

69	64	F	Stomach	NA	Surgery	Subsequent treatment strategy
70	81	M	Pancreas	WD, grade 2, Ki 67 8.6%, stage IV, NF	Surgery	Subsequent treatment strategy
71	69	M	Pancreas	WD, grade 1, Ki 67 1%, stage IV,NF	Surgery	Subsequent treatment strategy
72	70	F	Pancreas	WD, grade 1, Ki 67 < 3%, pT2N0M0, NF	None	Subsequent treatment strategy
73	47	F	Appendix	WD, grade 1, pT4N0M0, NF	None	Initial treatment strategy
74	77	F	Small Bowel	NA	Surgery	Subsequent treatment strategy
75	75	F	Lung	NA	None	Initial treatment strategy
76	91	M	Unknown Primary	WD, grade 2, Ki 67 15%, NF	None	Initial treatment strategy
77	67	M	Lung	WD, grade 1, Ki 67 1%, stage IV, F	None	Initial treatment strategy
78	75	M	Pancreas	NA	None	Subsequent treatment strategy
79	68	F	Small Bowel	WD, grade 2, Ki 67 12%, stage IV, NF	None	Initial treatment strategy
80	61	F	Pancreas	WD, grade 1, Ki 67 3%, stage II, NF	None	Initial treatment strategy
81	74	F	Small Bowel	WD, grade 2, stage IV, NF	None	Initial treatment strategy
82	67	M	Pancreas	WD, grade 2, Ki 67 3%, F	None	Initial treatment strategy
83	56	M	Pancreas	NA	None	Subsequent treatment strategy
84	62	M	Unknown Primary	NA	Surgery	Subsequent treatment strategy
85	55	M	Pancreas	WD, grade 1, Ki 67 0.8%, stage 1, NF	Surgery	Initial treatment strategy
86	64	F	Duodenum	NA	None	Subsequent treatment strategy
87	71	M	Lung	NA	None	Initial treatment strategy
88	54	F	Pancreas	WD, grade 1, Ki 67 2 %, stage II, NF	None	Initial treatment strategy
89	74	F	Small Bowel	Wd, grade 1, stage IV, NF	None	Subsequent treatment strategy
90	49	F	Unknown Primary	NA	None	Subsequent treatment strategy
91	44	M	Unknown Primary	NA	None	Subsequent treatment strategy
92	57	M	Pancreas	NA	None	Initial treatment strategy
93	85	M	Unknown Primary	WD, grade 2, ki 67 6.2%, NF	Chemotherapy	Subsequent treatment strategy
94	55	F	Unknown Primary	WD, grade 1, Ki 67 < 2%, pT2N0M0, NF	None	Subsequent treatment strategy

METs: metastatic; NF: non-functional; F: functional; NA: not available; WD: well differentiated, PR: poor differentiated
